# DOW-PR DOlphin and Whale Pods Routing Protocol for Underwater Wireless Sensor Networks (UWSNs)

**DOI:** 10.3390/s18051529

**Published:** 2018-05-12

**Authors:** Zahid Wadud, Khadem Ullah, Sajjad Hussain, Xiaodong Yang, Abdul Baseer Qazi

**Affiliations:** 1 Department of Electrical Engineering, Capital University of Science and Technology, Islamabad 44000, Pakistan; zahidmufti@nwfpuet.edu.pk; 2 Department of Computer Systems Engineering, University of Engineering and Technology, Peshawar 25000, Pakistan; 14pwcse1224@uetpeshawar.edu.pk; 3 School of Engineering, University of Glasgow, Glasgow G12 8QQ, UK; sajjad.hussain@glasgow.ac.uk; 4 School of Electronic Engineering, Xidian University, Xi’an 710071, China; 5 Department of Software Engineering, Bahria University, Islamabad 44000, Pakistan; abq.buic@bahria.edu.pk

**Keywords:** underwater wireless sensor networks (UWSNs), dolphin and whale pods routing (DOW-PR), end-to-end delay (E2ED), potential forwarding nodes (PFNs), suppressed nodes

## Abstract

Underwater Wireless Sensor Networks (UWSNs) have intrinsic challenges that include long propagation delays, high mobility of sensor nodes due to water currents, Doppler spread, delay variance, multipath, attenuation and geometric spreading. The existing Weighting Depth and Forwarding Area Division Depth Based Routing (WDFAD-DBR) protocol considers the weighting depth of the two hops in order to select the next Potential Forwarding Node (PFN). To improve the performance of WDFAD-DBR, we propose DOlphin and Whale Pod Routing protocol (DOW-PR). In this scheme, we divide the transmission range into a number of transmission power levels and at the same time select the next PFNs from forwarding and suppressed zones. In contrast to WDFAD-DBR, our scheme not only considers the packet upward advancement, but also takes into account the number of suppressed nodes and number of PFNs at the first and second hops. Consequently, reasonable energy reduction is observed while receiving and transmitting packets. Moreover, our scheme also considers the hops count of the PFNs from the sink. In the absence of PFNs, the proposed scheme will select the node from the suppressed region for broadcasting and thus ensures minimum loss of data. Besides this, we also propose another routing scheme (whale pod) in which multiple sinks are placed at water surface, but one sink is embedded inside the water and is physically connected with the surface sink through high bandwidth connection. Simulation results show that the proposed scheme has high Packet Delivery Ratio (PDR), low energy tax, reduced Accumulated Propagation Distance (APD) and increased the network lifetime.

## 1. Introduction

Underwater Wireless Sensors Networks (UWSNs) have numerous applications in diverse fields such as submarine detection, disaster prevention, oil/gas spill monitoring, off-shore exploration, and military target tracking. The characteristics of UWSNs e.g., high error rate probability, large propagation delay, floating node mobility and low communication bandwidth are totally diverse compared to terrestrial networks. Unfortunately, due to the dynamic nature of links, the connectivity of the sensor nodes cannot be assured and the reasons include rapid changes in network topology, variations in temperature, multi-path fading, environmental noises and interferences [1]. Since radio waves don’t have the ability to propagate through the water medium over long distances, sensor nodes use acoustic signals for communication. However, the presence of salt in seawater significantly affects the speed of sound. The average speed c of the acoustic wave fluctuates between 1450 m/s to 1550 m/s, which depends on the temperature and pressure in water. Depth Based Routing protocol (DBR) [2] uses the depth information of the sensor node for flooding the data packets towards the centralized station. The depth can be found with the help of depth sensor, which is integrated within every sensor node. The flooding is omni-directional so any node that is in the range of a sensor receives the packet. The sensor node adds its depth information to the packet. This depth information is compared by the receiving node with its own depth. In case the current node is shallower than the depth information appended in the packet, the receiving node is a PFN. The PFN holds the packet and sets the timer based on the holding time computation. In case PFN does not receive any duplicate copy of the packet until the expiration of the timer, it will forward the packet. On the contrary, if the node receives a duplicate copy of the packet before the expiry of the timer, then it will simply drop the packet. In case the receiving node is deeper than the sender node, it will drop the packet if and only if there is no PFN available to source. Weighting Depth and Forwarding Area Division protocol (WDFAD-DBR) [3] will choose the forwarder node by calculating the weighting sum of the difference in the depth at two hops. The DBR only considers the first hop PFNs for data forwarding but on the other hand WDFAD-DBR uses the accumulative depth at two hops nodes. Our proposed scheme, Dolphin and Whale Pods Routing (DOW-PR) protocol considers the number of PFNs, number of suppressed nodes and the hop count to select the node for forwarding the packet generated/forwarded by the source node. Nonetheless, the proposed scheme will select the shallowest suppressed node for forwarding the packet if the source node suffers from void region towards the sinks. The proposed scheme will divide the transmission range into different energy levels, so that the node (if selected as forwarder) that is closer to the source node will require less amount of transmission energy compared to the node that is far away. Therefore, the transmission energy will not remain constant through the transmission range; rather, it will vary in different energy levels. Furthermore, we propose another scheme called whale-pod comprised of multiple sinks placed at the water surface with an additional sink deployed underwater at the depth of 700 m. A high bandwidth physical connection exists between the embedded sink and the surface sinks. When the packet is received by the embedded sink, it is considered as a successful delivery of a packet to the destination.

Contributions: The contributions of our work have been summarized in the itemized text below: (1) The optimal set of mapped values for number of potential forwarding nodes and number of suppressed nodes has been investigated; (2) Significant energy is saved due to optimal route discovery mechanism; (3) Additional energy conservation achieved by dividing forwarder region into transmission power levels; (4) An optimal solution provided to cater for the problem of voids and energy holes; (5) Performance parameters included in formulating the holding time i.e., number of potential forwarder, number of suppressed nodes, hop count; (6) Significant improvement in end-to-end delay achieved by readjusting the position of sink nodes; (7) Traffic congestion sorted out by averaging potential forwarding nodes which forms the basis of item 2. To implement the above mentioned contributions, we follow the following steps:

- Selection of forwarder by computing the optimal average number of PFNs of the forwarding nodes,
- Calculating optimal transmission power adjustment based upon more distant node from the source node in potential forwarding region,
- Finding the alternate node from the suppressed region for the case if source node is in a void,
- Carrying out packet holding time calculations to assign priorities,
- Finding the nearest sink for the case if one of the sink is embedded underwater.

The rest of the manuscript has been structured as follows. The detailed discussion regarding previous work on routing protocols in UWSNs is carried out in Section 2. Section 3 is about the identification of the problem and present the problem statement. The system model is explained in Section 4. The experimental setup and simulation outcomes of our approaches are described in Section 5. Performance comparison and analysis discussed in Section 6. Finally, a brief conclusion is presented in Section 7.

## 2. Previous Work

In this section, we first review some related works on routing protocols in Underwater Acoustic Sensor Networks (UASNs), and will discuss their pros and cons. Then, we discuss the differences between WDFAD-DBR and proposed DOW-PR routing protocol.

The most important and recognized property of Wireless Sensor Network (WSNs) is Energy efficiency. There are many types of terrestrial WSN protocols i.e., Directed Diffusion [4], Two-Tier Data Dissemination [5], Gradient [6], Rumor routing [7], and Sensor Protocol Information via Negotiation (SPIN) [8], etc. The existing protocols designed for WSNs are not feasible for UWSNs because of multiple reasons that include high propagation delays, high mobility of nodes, severe multipath phenomena, acoustic signaling, etc. Numerous UWSNs protocols are proposed, and it includes, Depth Based Routing (DBR) [2], Vector Base Forwarding (VBF) [9], Focused Beam Routing (FBR) [10], Hop by Hop Vector Based Forwarding (HH-VBF) [11], Adaptive Hop by Hop Vector Based Forwarding (AHH-VBF) [12], Directional Flooding Routing DFR [13], Delay Tolerant Network DTN [14], etc. These networks have been proposed in the last few years for increasing energy efficiency in underwater WSNs. In UWSNs, some protocols are designed to achieved a higher efficiency in energy such as HH-VBF, AHH-VBF and DFR.

Routing protocols for UWSNs are categorized into four classes. The first such class is that of Flooding Based Routing (FBR) protocols, which includes HH-VBF, FBR, DBR, H2–DAB, DFR, etc. The second is multipath based routing, which include protocols presented by Winston et al. [15], Dario Pompili et al. [16] and so on. Third is Clustered Based Routing protocols, which include Minimum-Cost Clustering Protocol (MCCP) [10], Distributed Underwater Clustering Scheme (DUCS) [17], and Hydro Cast [18], etc. The last class being that of Miscellaneous Routing protocols including ADAPTIVE, Information-Carrying based Routing Protocol (ICRP) [19], Phero-Trail and so on.

Geographic based routing protocol such as VBF, HH-VBF requires a location information of the network where the nodes are deployed to send a packet to the centralized station from the source node. In VBF [9], a routing vector (a vector drawn between source and sink node) and a desirableness factor (suitableness) is defined so that the node that is close to the virtual vector and also to the sink node will be selected as a forwarder. HH-VBF is proposed in contest of VBF. In HH-VBF, instead of using fixed virtual vector and fixed forwarding pipeline radius, it is redefined at each hop in the whole network lifetime. The reliability and PDR of the network is improved due to this amendment in VBF but at the cast of increase in communication overhead. In uneven distributed networks (i.e., sparse networks, where the number of nodes is less), the performance of HH-VBF is lower than that of VBF. The reason behind this drawback is the constant pipeline radius maintained throughout the network lifetime.

In [12], Hwang et al. proposed AHH-VBF. In order to restrict the forwarding range and improve the PDR, AHH-VBF changes the width of pipeline, according to network nodes distribution, in order to control the forwarding area. Unlike HH-VBH, AHH-VBF can adaptively adjust the power level hop by hop according to the neighbor node distribution of local region. Therefore, energy consumption is reduced and lifetime of the network is increased.

ESEVBF (Energy Scaled and Expanded VBF) [20] scales and expands the holding time with the residual energy of all forwarding nodes in the potential forwarding zone (PFZ). The ultimate goal achieved in this protocol is to reduce the duplicate packets due to the imbalance between the holding time difference and propagation delay. Consequently, it reduces the energy expenditure of the network along with considerable improvement seen in end-to-end delay. However, it does not provide any improvement in PDR compared to counterpart AHH-VBF.

In FBR [10], the unnecessary flooding (i.e., the forwarding of a packet by a router from any node to every other node attached to the router except the node from which the packet arrived) was reduced where the transmission power constrained the flooding. FBR uses different transmission power levels that range from P1 to PN, in order to reduce the consumption of energy in UWSNs, so that life of the network is enhanced. The FBR is a location based protocol where each node knows its own location and that of the sink. In FBR, the transmission radius, corresponding to each power level, is the area within a cone of an angle originating from the source node towards the sink. FBR uses two packets request to send (RTS) and clear to send (CTS) in the next forwarder selection scheme. To select PFN, source node broadcasts an RTS packet with a power level P1. In response to the RTS packet, the PFNs initiate the CTS packet. The CTS packet(s) received by the source node. In case, if more than one CTS packet received by the source node, then it will select the PFN as a forwarder that is closest to the destination. For the case, if no CTS packet is received from any PFN, then the source node boosts its power level to find more PFNs. The process repeated until the source node finds PFNs. When power level becomes PN and still no CTS packet is received by the source, then the cone is shifted to any direction around the main cone. In this way, the packet is transmitted from a source to a sink.

In DFR [14], the author proposed a novel protocol that improves the efficiency of UWSNs, where the source node and sink node determine the base angle so that the flooding region of the packet is confined. If a node receives a packet, it will first check whether the node is within the base angle and then it will forward the packet; otherwise, directly discard it. DFR controls the range and the direction of flooding packet due to which a great deal of energy is saved. However, the problem with DFR is the consistent transmuting power amplitude maintained regardless of whether the forwarder is placed near or far. For this very reason, the protocol suffers from energy unbalancing among the network nodes. Additionally, in sparse networks, it is difficult to find out the PFNs due to more probability of void hole occurrence.

Another routing protocol belongs to Communication path based routing protocol, whose scheme is abbreviated as Relative Distance Based Forwarding (RDBF) and is introduced in [21]. This scheme emphasizes end-to-end delay, decrementing wastage of energy and the efficient routing mechanism. The delay and wastage of energy can be minimized if less number of nodes will participate in data packets forwarding. RDBF also implement the mechanism look after the problem of duplicate packets to transmission time adjustment of forwarding nodes.

A clustering scheme is used in [22,23] for routing in UWSNs, which consists of two phases i.e., set-up phase and communication phase. The cluster heads are selected in set-up phase, while, in the communication phase, the nodes collect the data and forward it to their corresponding clustering heads. However, as the nodes move more frequently in the horizontal direction and very little in the vertical direction, there will be water currents all the time and the clustering head selection is repeated, which produces more overhead on the network. After this, the nodes collect and forward the data to the sink node only when all the clustering members collected the data, which results in long end-to-end delays. Therefore, clustering schemes are not good for UWSNs, especially when there are real-time applications that are time intensive too, while, for time critical applications, a novel protocol is proposed for UWSNs called multipath routing protocol in [24]. However, due to the water current, the topology of the network may change and the path calculated in the previous round is not optimal in the current round. In addition, when we transmit the available packets, a lot of energy is wasted and each time source node requests to establish a multi-paths or optimal path according to the bit error rate, propagation distance and energy consumption of each link.

Two types of holes are created in wireless sensors network, one is a void hole that occurs due to unavailability of forwarder nodes, and the other is an energy hole that occurs due to the imbalanced data traffic in the network. The creation of a hole is a major factor in degrading the performance of the entire network. In this regard, Zahid et al. [25] proposed a protocol to maximize the network lifespan via hole alleviation. It evenly divides the number of transmissions over various network areas that are capable of balancing energy dissipation among the nodes.

Sherif et al. [26] authors introduces delay tolerant network (DTN) routing protocol, which is about the motion of node, to handle continuous movements of nodes and to make practical and effective use of the single-hop and multi-hop routing schemes.

The objective of these two routing protocols i.e., Round-Based clustering (RBC) [27] and Link-State Based routing (LSB) [28] is to minimize energy utilization or wastage by sensing nodes. These routing protocols give us different possible solutions so that energy can be saved in the sensing nodes at the physical layer, medium-access control (MAC) and routing layers. Such routing protocols consider the conditions of water, whether it is deep or shallow water, as the parameters of both water conditions are totally different. The big problem faced by the MAC layer is the larger overhead to mitigate packet collision rate. This collision overhead can also be tackled by the routing protocols. In order to remove redundancy of data packets in the network MAC layer, solutions to overcome the confines are proposed. This routing protocol uses rounds, and each round has four different stages; in these four stages, this scheme utilizes an appropriate mechanism in order to resolve the issue. The proposed clustering routing protocol minimizes the energy consumption of the network, which in turn enhance throughput and lifespan of the network.

Wan et al. propose PSFQ (Pump Slowly and Fetch Quickly) [29], in which hop by hop mechanism is utilized. The theme is that the sensing node forwards the data unit close to the neighbors with delay or with less speed. If any data packet has been lost, the receiver immediately gets that data packet that has been lost in the acoustic sensors’ network channel. The receiver gets the packet that has been lost more frequently at a rate five times faster than the sender node that sends the data packet to its immediate neighbor. Data packets are sent towards the sink node passing through multiple hops in a consistently good manner and with reliability. In PSFQ, ARQ (Automatic Repeat Request) utilizes a hop-by-hop mechanism. However, due to one of the intrinsic characteristics’ long propagation delay caused due to acoustic signals, ARQ is unable to utilize more acoustic channels in UWSNs.

Another routing protocol or routing scheme is Adaptive Mobility of Courier nodes in Threshold-optimized Depth-based (AMCTD) [30]. This proposed routing protocol enhances the lifespan of the entire network. The assumptions that are used in this routing protocol are very concerned with and helpful in the reduction of energy wastage of the sensing nodes having low depth during the stability period. It makes available the computation of weight with optimization, balances the network globally, and also provides holding time computation efficiently for the sensing nodes that send the data packets to its neighbors. AMCTD also enhances the lifetime of the entire network and increments the stability of the whole network while deploying the courier nodes efficiently as well. It proposes a function called efficient Weight Functions (WF), which is used to increment the stability duration of the sensors network. This scheme also provides a model in order to decrement noise, path losses and other attenuation factors for the sensors that are present at the region in UWSNs where the depth is less.

H2-DAB protocol, which is also abbreviated as hop by hop dynamic addressing based protocol, is free of location information, as it belongs to a beacon based routing protocol class, as localization information of the node is costly, so, in this way, it reduces the cost of hardware. This scheme counts the hop number from the sink nodes present on the surface, and assigns a distinct address to all sensors that are part of the network by using the beacon mechanism from the nodes present on the surface (sink nodes). The node that has larger dynamical and distinct address will forward the packet towards the node that has a dynamically smaller address, up until it reaches the destination node or sink node. In [31], this scheme is used by using the concept of unique IDs of the sensing nodes. If it is assumed that packets are forwarded towards the sinks, then multi sink architecture must be taken into consideration in case any sink node detected the data packet successfully.

In [32], mutilpath routing protocol is introduced in which it is considered that a source node sends a multipath route request towards the destination node before it sends the data packet to the destination. In response to the request, multiple paths/routs are created between source and destination. On the basis of two parameters, the source node sorts out the optimal route. Those two parameters are path length (number of hops) and energy distribution factor along this paths. Source node selects power for the transmission and will send the data packet through that path in which energy is evenly and fairly distributed. The node that is present at the destination will receive redundant data packets as repetition of packets due to multiple paths. It is totally dependent upon the destination node to choose one fault free packet and discard the others. In a multipath based routing scheme, more energy will be consumed because of redundancy of packets as the data packets received through different paths.

The authors in [2] proposed DBR in which the information about the full dimension and location of a node is not required. DBR is based on the depth information, which can be easily found by a depth sensor integrated within every sensor node to forward a packet from the source node to the sink. DBR uses a greedy mechanism to find one hop forwarding path. However, the selected forwarding node may not be able to forward the packet due to occurrence of void hole, which reduces the PDR.

In [33], the authors propose an Energy-Efficient Depth-Based Routing scheme (EEDBR) to enhance the life of the network and stability period of the network by considering the depth and the energy remaining in the nodes. EEDBR achieved the significant improvement E2ED along with the better utilization of the energy bank, especially for nodes that are shallower. EEDBR protocol implements the phenomenal through formulating the holding time and that depends upon current energy storage level. There is a lack of energy fairness because energy is unevenly distributed among sensors. Further, a detailed qualitative comparison of different characteristics of underwater sensor networks is provided in Table 1.

First and foremost, the problem of void holes (absence of forwarder in the transmission range of the source node) occurrence is exploited by the authors in our baseline paper i.e., WDFAD-DBR [3]. The design of the algorithm ensures the significant reduction of the packet-drop due to successful void holes mitigation, especially when the network is sparse. WDFAD-DBR considers the depth of the forwarders at the immediate and next hop. The weighting value is assigned to potential forwarders at each hop in such a way that maximum advanced distance can be achieved. The priority of the two-hop path selection effectively overcomes the frequent upward hemispherical void regions. The objective function implemented through the design of the holding time calculation is based upon the weighting sum of the parameters i.e., first and second hop depths. Secondly, the baseline protocol WDFAD-DBR also achieves better conservation of the network energy usage. The run-time readjustment of the forwarding area division encounters multiple copies of packet propagation in the network. To support the above phenomenon, the WDFAD-DBR will judge the link conditions and sparseness or denseness of the nodes in the network. The prediction of neighbors are implemented in accordance with the mobility pattern, speed and *x*, *y*, *z*-coordinates among nodes. Thirdly, the collision of the packets is investigated while contending the channel at the MAC layer.

In this paper, our proposed protocol DOW-PR focuses on selecting the optimal forwarder. This is very similar to the WDFAD-DBR. Much like WDFAD-DBR, DOW-PR also considers the weighting sum of depth of the current and the next expected hops’ sensor nodes. The novelties of the proposed protocol that differentiate themselves from counterpart WDFAD-DBR is mentioned in itemized text as follows:

- To improve the performance of WDFAD-DBR, we propose a state-of-the-art DOW-PR routing protocol in which we divide the transmission range into different transmission power levels while selecting the next forwarding node. The source node searches for the optimal power level for packet transmission.
- We also consider the additional parameters i.e., number of PFNs and number of suppressed nodes. WDFAD-DBR does not consider the above-mentioned parameters due to which a network consumes a significant amount of receiving energy, especially in dense networks.
- Along with other parameters, our scheme also considers the number of hops traversed by the packet initiated from the source node. Consequently, DOW-PR optimizes the shortest possible path and thereby improves the end-to-end delays.
- WDFAD-DBR does not provide any mechanism for void hole occurrences at the second hop forwarder. Our proposed protocol DOW-PR will select the node for broadcasting from the suppressed nodes when there is no PFN available.
- In DOW-PR, we also propose another system (Whale pod) in which multiple sinks are placed at water surface, but only one sink is embedded inside the water and will be physically connected with the surface sink through high bandwidth connection.

## 3. Problem Statement

**Figure 1:**
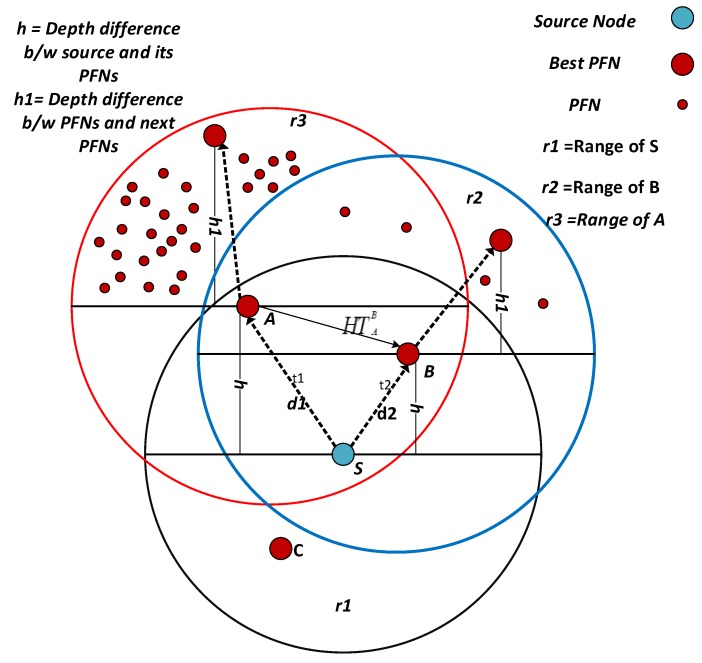
Forwarder node selection scenario.

The WDFAD-DBR protocol abbreviates the priorities of PFNs in designing the holding time of the received packets by considering the accumulative depth differences of the potential forwarding nodes at hops 1 and 2 [3]. WDFAD-DBR does not only consider the depth of the current node, but also the depth of the node at the next expected hop. Therefore, weighting sum of the depth difference i.e., *H* is the combination of depth difference *h* between the source node and next PFN and the depth difference *h1* between the PFN at hop 1 to the next expecting PFN at hop 2. However, WDFAD-DBR does not consider the number of suppressed nodes and number of PFNs of a source node, which consumes a significant amount of receiving energy. The reason behind it arises from the fact that a large number of PFNs result in receiving the packet as well as the high probability of duplicate packets generated at the first hop and hence excessive transmission energy wasted. The second important reason is that WDFAD-DBR does not consider the hops number for a packet to travel through. In case of a void hole i.e., when the forwarding node does not exist or the existing forwarding node does not have enough energy to communicate, WDFAD-DBR will drop the packet straight away and therefore Packet PDR degraded.The proposed protocol considered the number of suppressed nodes, number of PFNs, and hop count of each potential forwarder as well as the weighting sum of the two hops neighbors. For instance, if considering node S as the source node and nodes A and B are the next hop potential forwarding nodes, as shown in Figure 1. WDFAD-DBR will select node A as a forwarding node, as weighting sum of heights for two hops is greater than any other path. However, node A having a large number of PFNs will suffer from a large amount of receiving and transmitting energy due to the chance of initiating duplicate packets.The causes of duplicate packets has been discussed in Section 3.2. The proposed dolphin-pods routing will give preference to node B for forwarding in order to overcome the above-mentioned problems. When we consider the number of PFNs and number of suppressed nodes, a reasonable amount of receiving energy will be conserved. Moreover, WDFAD-DBR considers the fixed transmission power level for all nodes in the range of a source node, whereas the proposed scheme divides the transmission range into different transmission power levels such that the appropriate transmission energy level is used by the source node to conserve the energy.

### 3.1. Preliminaries

The following notations are used in our proposed DOW-PR scheme:

- *Sink Node D*: A UWSN sink node (also called destination node) is a type of node that is placed at the ocean surface or embedded inside water. Primarily, its function is to collect data from the sensor node and forward it to the base station through high speed radio link. These sinks or destination nodes can be static or mobile. Let D be a set of network sinks, then:
  (1) $$D=(D_{1},D_{2},D_{3},D_{4},\ldots \ldots ,D_{8},D_{EM}).$$
- *Transmission Range ($T_{r}^{S}$) of Node S.* Transmission range of node S is an omni-directional distance from source node S($x_{s}$, $y_{s}$, $z_{s}$) that currently forwarded the packet p until where it can transmit the packet p.
- *Eligible Neighbors ($EN_{i}$) of Node i*: Nodes that are in transmission range of a node i. Let N be a set of nodes in a network
  (2) $$N=\{n_{1},n_{2},n_{3},n_{4},\cdots ,n_{k}\}.$$
  Then, Eligible Neighbors of Node i can be expressed as $EN_{i}$⊆ N
  (3) $$EN_{i}=\left\{j\in N\;\wedge \;DIST_{j}^{i}\leq T_{r}^{i}\right\},$$
  where $DIST_{j}^{i}$ is the Euclidean distance between node i ($x_{i}$, $y_{i}$, $z_{i}$) and node j ($x_{j}$, $y_{j}$, $z_{j}$) in three-dimensional Euclidean space:
  (4) $$DIST_{j}^{i}=\sqrt{{\left(x_{i}-x_{j}\right)}^{2}+{\left(y_{i}-y_{j}\right)}^{2}+{\left(z_{i}-z_{j}\right)}^{2}}.$$
- *Potential Forwarders ($PF_{i}$) of Node i*: Potential Forwarders of node i are those nodes that are in transmission range $T_{r}^{i}$ and their depth ($d_{j}$) is less than depth ($d_{i}$):
  $$PF_{i}\subseteq EN_{i},\text{ where}$$
  (5) $$PF_{i}=\left\{j\in EN_{i}\;\wedge \;d_{j}\leq d_{i}\right\}.$$
- *Potential Forwarding Zone (PFZ)*: Potential Forwarding Zone (PFZ) is the hemispherical region whose radius is equal to $T_{r}^{S}$ and each point in PFZ has lesser distance to the sink as compared to source node. PFZ is the subregion of $T_{r}^{S}$ of node S and the nodes in the region are called potential forwarder nodes (PFNs), which are next forwarders of packet p. Any point in 3D Euclidean space q($x_{q}$, $y_{q}$, $z_{q}$) is considered to be in the PFZ of S, if it satisfies the following conditions:
  $$DIST_{D}^{q}<DIST_{D}^{S},DIST_{S}^{q}<T_{r}^{S},\text{ where}$$
  **a**  $DIST_{D}^{q}$ is the Euclidean distance between point q($x_{q}$, $x_{q}$, $x_{q}$) and Sink D($x_{D}$, $y_{D}$, $z_{D}$) in three-dimensional Euclidean space:
    (6) $$DIST_{D}^{q}=\sqrt{{\left(x_{q}-x_{D}\right)}^{2}+{\left(y_{q}-y_{D}\right)}^{2}+{\left(z_{q}-z_{D}\right)}^{2}}.$$
  **b**  $DIST_{q}^{S}$ is the Euclidean distance between point q($x_{q}$, $x_{q}$, $x_{q}$) and Source S($x_{s}$, $y_{s}$, $z_{s}$) in three-dimensional Euclidean space:
    (7) $$DIST_{q}^{S}=\sqrt{{\left(x_{s}-x_{q}\right)}^{2}+{\left(y_{s}-y_{q}\right)}^{2}+{\left(z_{s}-z_{q}\right)}^{2}},$$
    $$Z_{q}\leq Z_{s}.$$
  Neighbors of node i that are in PFZ of S:
  (8) $$X_{i}=\left\{n_{i}\in PF_{i}\;|\;DIST_{n_{i}}^{i}\leq T_{r}^{i}\wedge \;Z_{n_{i}}\leq Z_{s}\right\}.$$

### 3.2. Causes of Duplicate Packets

Primarily, the duplicate packets are generated due to the following facts:

- Firstly, the holding time of packet p at node i $HT_{i}^{p}$ is computed by a node i and the timer is started upon successful reception of packet p (refer to Figure 1). Node i does not forward the packet when $HT_{i}^{p}$ is on, however, data packets from neighboring nodes can be received by it, which may be duplicates of p or other data packets. Before the expiry of $HT_{i}^{p}$, if node i receives additional copies of p (a single or multiple copies), it abandons the transmission of p. However, for the case that no copies of packet p are received before $HT_{i}^{p}$ expiry, packet p is forwarded by i. Hence, simply by duplicating broadcast overhead is minimized, which is essential when bandwidth and energy are scarcely available resources as in UASN scenario. However, if in case, the holding time difference between any two nodes A and B ($HT_{A}^{p}-HT_{B}^{p}$) is smaller than the propagation delay of a packet p from node A to B, the duplicate packets will be generated.
- The second reason for generating the duplicate packet is the hidden terminal problem. In a hidden terminal problem, the source node broadcasts and the potential forwarding nodes receive the packet. The problem occurs when the highest priority node broadcasts the packet while some of the potential forwarding nodes of the source node are not in the range and thus do not receive the duplicate packet, which causes these packets to be generated.
- Thirdly, relaying packets over multiple hops might result in a failed delivery of the packet to its destination because of high error rate of the acoustic channel, path losses and channel impairments. Duplicate packet generation and transmission become imperative because of the above-mentioned scenarios.

## 4. Proposed Scheme

In this section, we describe the network architecture, division of transmission range in different transmission power levels, and selection of suppressed node in the absence of potential forwarding nodes.

### 4.1. Network Architecture

**Figure 2:**
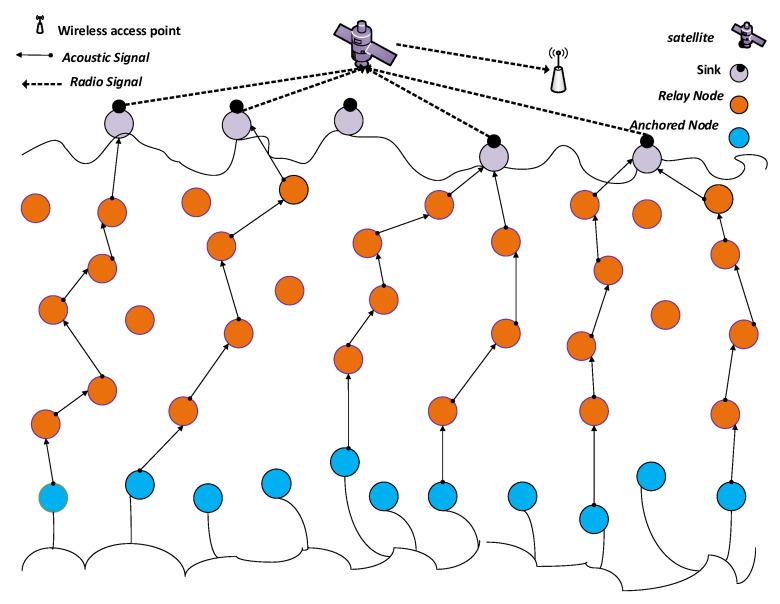
Network architecture.

The network architecture of DOW-PR protocol is composed of sink nodes, relay nodes and anchored node as shown in Figure 2. Sink stations are situated at the sea roof and consists of radio and acoustic modem in order to communicate with each other through radio link and with the sensor networks through acoustic signals. These nodes are centralized stations, which can receive and transmit signals to the external networks. Anchored nodes are fixed at the seabed and their task is to collect data from the environment. Anchored nodes are fixed with the tether [34] and movable with water current or any other disturbance in the environment. Relay nodes are deployed at different depths, which forward the received data. Sink nodes can communicate within water through acoustic links and communicate with the external network through radio links. Basically, sink nodes are the centralized stations. Since sink nodes can communicate with each other, the data packet received by any sink nodes will be considered a successful delivery to the destination. Typical applications of this network include monitoring of underwater plates in tectonics or environmental monitoring [35].

### 4.2. Acoustic Signal Velocity in the Underwater Environment

**Figure 3:**
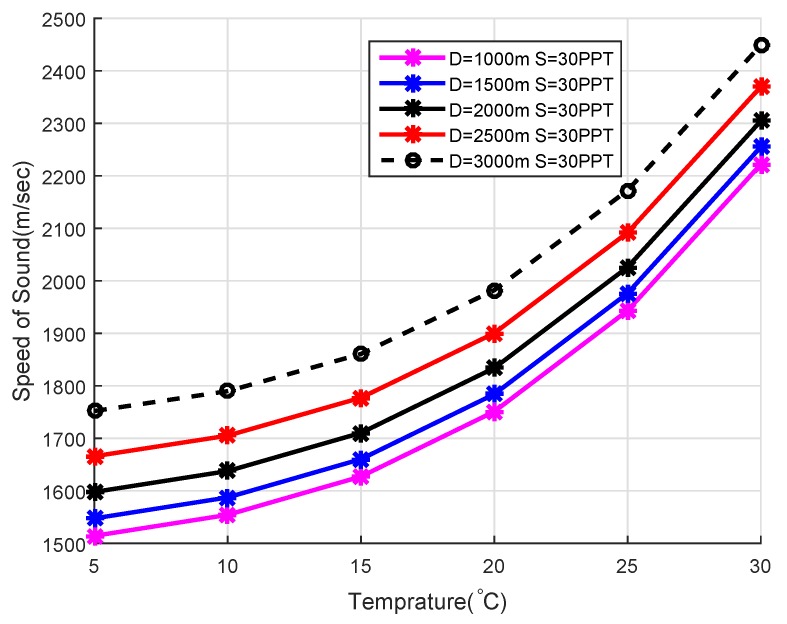
Speed of sound vs. temperature.

**Figure 4:**
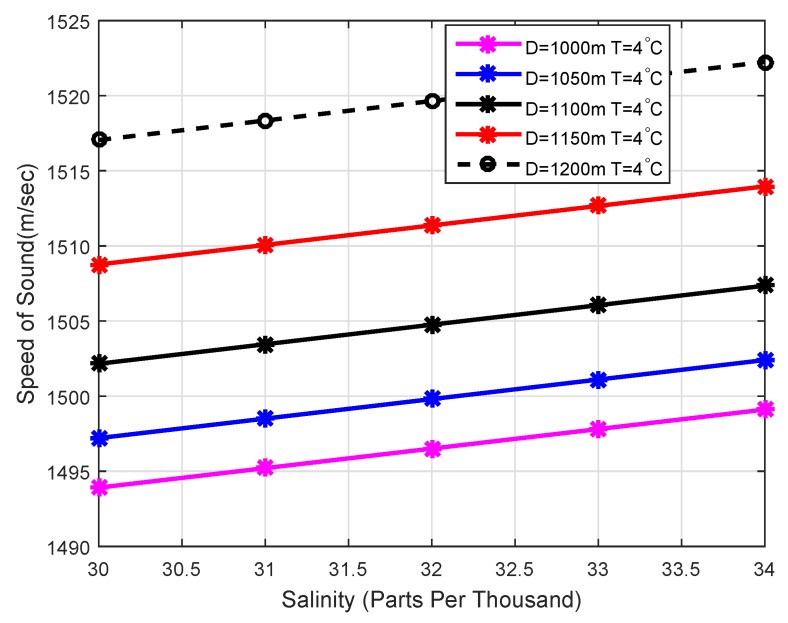
Speed of sound vs. salinity.

Different factors affect the speed of acoustic waves i.e., temperature variation, pressures at different layers of the sea and salinity of the water. Mathematically, it can be related as [36]: $c=1446.96+4.591T-5.305\times 10^{-2}T^{2}+2.374\times 10^{-2}$$T^{3}+1.340\left(S-35\right)+1.63\times 10^{-1}D+1.675\times 10^{-7}$$D^{2}-1.025\times 10^{-2}T\left(S-35\right)-7.139\times 10^{-13}TD^{3},$ where *c* represents the velocity of the acoustic signal in m/s , *T* represent the temperature in degree Celsius, *S* is the salinity in parts per thousand and *D* represents the depth in meters. The sound speed increases with the increase in temperature as shown in Figure 3 and speed of sound variation with respect to salinity shown in Figure 4. The above Equation is valid for 0 ${}^{\circ }$C ≤T≤ 30 ${}^{\circ }$C, 30≤S≤ 40 PPT, 0≤D≤ 8000 m.

### 4.3. Acoustic Signal Reflection/Refraction in the Underwater Environment

Channel geometry and its reflection and refraction properties influence the impulse response of an acoustic channel. The total count of major paths for propagation and their relative delays and strengths are also determined by these characteristics. Strictly speaking, the number of signal echoes is infinitely large, but after discarding those which have undergone multiple reflections and thereby lost much of their energy, we are left with only a few significant paths. The longest path delay governs the total multipath spread, which is to the tune of tens of milliseconds. Such values are usually reported in shallow-water experiments [37]. The dispersion of individual paths is significantly lesser than the total multipath spread. Therefore, for systems with maximum frequencies significantly below the channel cutoff (several tens of kilohertz in our simulations), it can be ignored. For systems currently in use, this is typically the case. For the sake of a fair analysis, we ignore the reflection phenomena for both DOW-PR and WDFAD-DBR in our simulations.

### 4.4. Energy Propagation Model

The acoustic channel attenuation in UWSNs over *d* is defined by the following formula [38]:

10logA(d,f)=k·10logd+d·10logα(f).

Two terms used in the above equation i.e., the spreading loss and absorption loss. *k* may have values i.e., 1, 1.5 and 2 for represents the spreading coefficient which defines the geometry of the propagation. If *k* = 1, the geometry of propagation is cylindrical spreading in shallow water, however *k* = 2 pertains to the geometry of the propagation spherically spreading in deep water, while, for *k* = 1.5, the geometry of the propagation is practical spreading where α(f) represents the absorption coefficient. The underwater noise can be found by the following expression:

$$N\left(f\right)=N_{t}\left(f\right)+N_{s}\left(f\right)+N_{w}\left(f\right)+N_{th}\left(f\right),$$

where $N_{t}\left(f\right)$ represents turbulence noise, $N_{s}\left(f\right)$ represents shipping noise, $N_{w}\left(f\right)$ represents waves noise and $N_{th}\left(f\right)$ represents thermal noise. Noise in acoustic channels comprises site-specific noise and ambient noise. While site-specific noise only exists in certain places, backgrounds of quiet deep seas always have ambient noise present. For instance, snapping shrimps in warmer waters create acoustic noise and so does ice cracking in polar regions. Turbulences, rain, breaking waves and distant shipping give rise to ambient noise as shown in the above equation. Although this noise is not white, it’s usually approximated as Gaussian. Contrary to ambient noise, significant non-Gaussian components are present in site-specific noise. Our simulation only considers narrow band ambient noise and that is taken to be Gaussian. Ambient noises’ acoustic signal is distorted due to ambient noises that have completely different effects depending upon the location and frequency. Practically, the noises discussed above are the major contributors at frequencies from 10 to 100 KHz.

Noise spectrum levels typically decrease from about 140 dB re 1 μPa${}^{2}$/Hz at 1 Hz to about 30 dB re 1 μPa${}^{2}$/Hz at 100 KHz . We have used a central acoustic signal frequency of 12 KHz with an approximate noise level of 50 dB re 1 μPa${}^{2}$/Hz to fair noise model close to practical values.

### 4.5. Packet Types in the Dolphin and Whale Pods Routing

There are three various types of packets in DOW-PR routing protocol, which are NEIGHBOR REQUEST, ACK and DATA. The source node uses packet *NEIGHBOR REQUEST* to search its qualified forwarding nodes. Its format is shown as an NR (TID, SID, DP, VA). TID field is a two-bit number that differentiates between the packets. The TID for NR is “00”. SID abbreviated as ID of the source and it is broadcast in the neighbor request message. DP represents the depth of source node and VA is a one bit number represents the void hole announcement. The value of VA will be true if the source found a void hole. *ACK* packet is sent in reply to neighbor request means the neighbor node send its information. The format of ACK is ACK (TID, SID, DP). The TID for ACK packet is “01”, SID presents the identification ID of the current neighbor sensor and DP is the depth of node sending *ACK* packet. *DATA* is the real data and it has header and payload. The format of DATA is (TID, SID, DID, DP, PID). The TID value for DATA packet type is “10”, SID is the source ID, DID represents the destination address, DP represents source depth and PID representing packet sequence number. The neighbor request and Acknowledgment packet has smaller size than the DATA packet.

### 4.6. Division of Transmission Range into Different Transmission Power Levels

**Figure 5:**
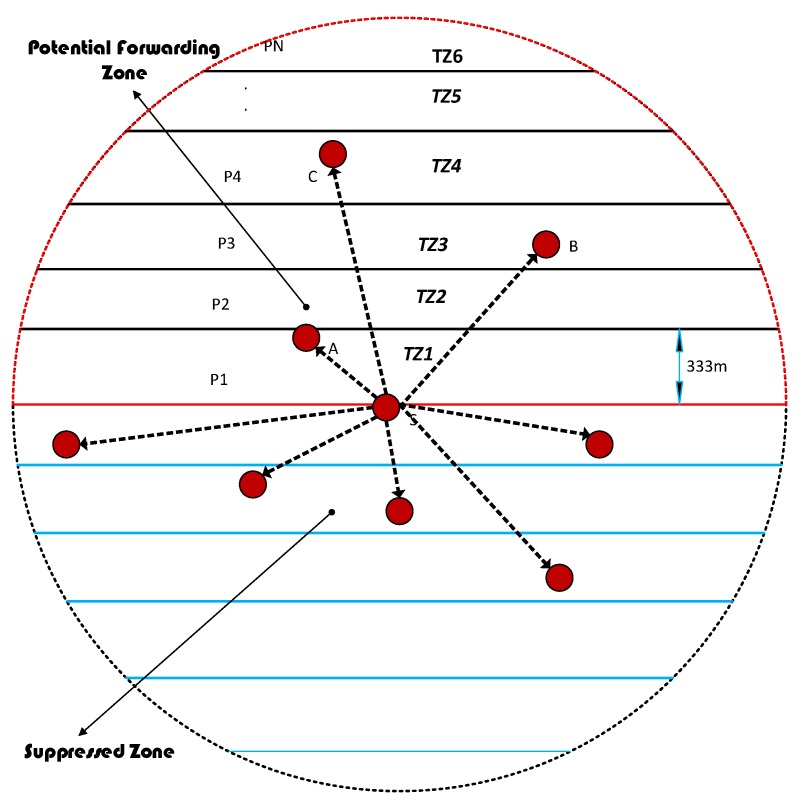
Division of transmission zones (TZ1–TZ6).

The proposed protocol divides the transmission range into six different transmission energy levels as shown in Figure 5. For example, the next forwarder is close to the source node i.e., in transmission zone TZ1, then it will require less transmission energy. On the other hand, if the next potential forwarding node is far away in transmission range from the source node i.e., in TZ6, then higher transmission energy will be required. To increase the network lifetime, the proposed scheme uses different transmission power levels, which range from P1 to PN for broadcasting a DATA packet. The sender node floods a neighbor request message using power intensity level of PN. All the neighboring nodes receive the neighbor request message and reply with an acknowledgment packet. According to the acknowledgment packets received from different neighboring nodes from different transmission levels, the source node sets the transmission power. For example, from Figure 5, the source node S broadcast a neighbor request with a power level PN. The node A in transmission zone TZ1, node B in TZ3 and node C in TZ4 level received the neighbor request. The nodes A, B, and C reply with the acknowledgment. The acknowledgment packet contains the depth field pertaining to the depth of the sender. According to the DP field in the acknowledgment packet, the source node found the nodes in different transmission levels. The node A is lying in transmission portion TZ1, node B placed transmission zone TZ3, while node C is in transmission zone TZ4. Thus, the node C has a smaller depth than all the other nodes. The source node sets the transmission power level to P4 for broadcasting the DATA packet and with this power level all the three nodes received the packet successfully. The nodes then calculate the holding time and set the timer according to their holding time.

### 4.7. Selection of a Forwarding Node among Suppressed Nodes

**Figure 6:**
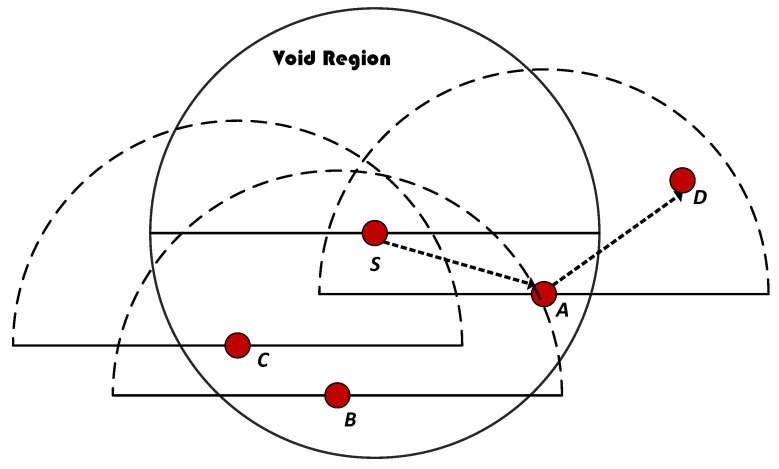
Forwarder selection from the suppressed nodes.

WDFAD-DBR selects the route based upon the weighting sum of depth difference between first and second hop PFNs. WDFAD-DBR drops the packet when there is no PFN(s) found and that means the data is lost. The source node S finds PFNs by sending a neighbor request packet. However, if the source node does not have any PFN, then the node for forwarding the packet will be selected from the suppressed nodes (refer to Figure 6). The selection of suppressed node will be based on the depth and having PFN(s) other than the source node. The source node S will select node A for forwarding, which has smaller depth in suppressed nodes and also has a PFN D, which then continues broadcasting, ensuring minimum lost data.

### 4.8. Holding Time Estimation

When neighbors of a source node receive data packets, it decodes and extracts the depth information of a source node and compares it with its own depth field DP. If DP value of the receiver is greater than DP value of the source node, and also the void announcement VA field has a value of 1, then it will forward the packet after necessary holding time calculation when no PFN is available to source. For the case, if PFNs are available, then each PFN will calculate the holding time according to the Fitness Function (HH) value, which is described below. The proposed scheme not only considers the sum of depth difference of the two hops (H), but also considers the number of PFNs ($PFN_{num}$), number of suppressed nodes ($SUP_{num}$), and the hop count from PFN to sink, which is best in favor of performance metrics. Thus, the proposed scheme will consider all of the above-mentioned factors in selecting the next forwarding node.

**Figure 7:**
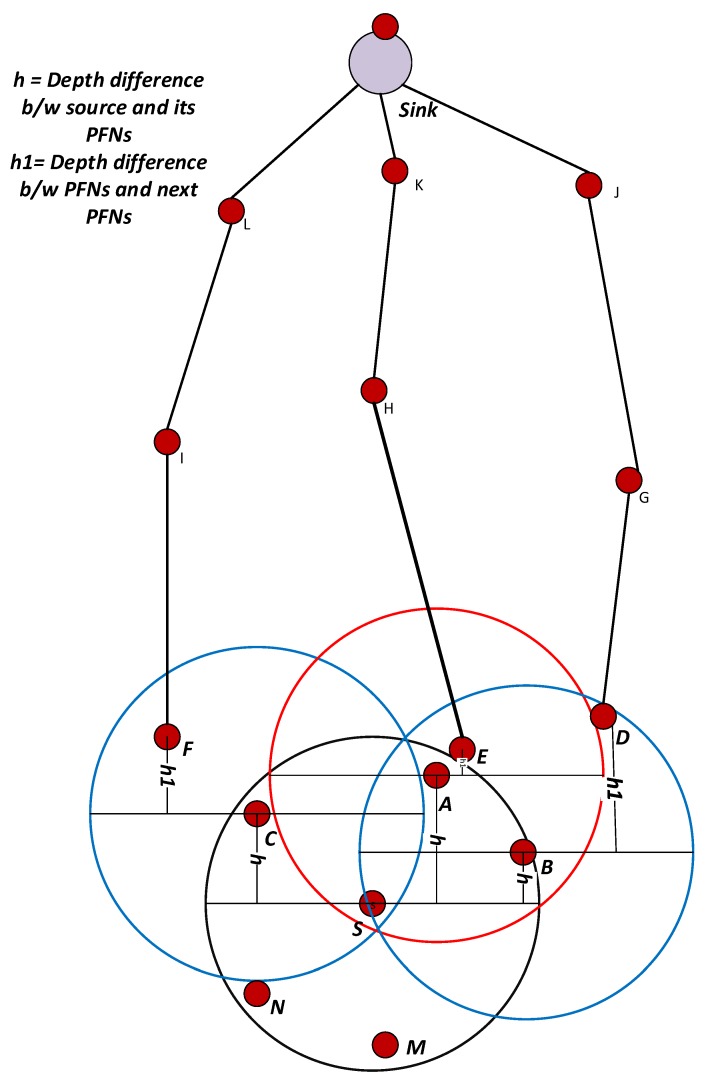
Holding time scenario.

To find the Fitness Function (HH) value, we mapped the $PFN_{num}$, $SUP_{num}$ into arbitrary values called as division factors represented by $DIV_{PFN}$ and $DIV_{SUP}$, respectively. This is further elaborated in the simulation analysis section:

(9) H=αh+(1−α)h1,

where *h* is the depth difference of the source node to its PFN and *h*1 is the depth difference of the PFN to the next expected hop and α is weighting coefficient and its value is between 0 and 1. For node A, h is the depth difference of the source node S and itself A and h1 is the depth difference of node A and E as shown in Figure 7. The Fitness Function is then calculated as:

(10) $$HH=\frac{H}{\left(\left(DIV_{PFN}+DIV_{SUP}\right)\times HOP_{tosink}\right)}.$$

The holding time is a function of the fitness value:

(11) T(H)=k∗(HH)+β,

(12) $$T\left(H\right)=k\frac{H}{\left(\left(DIV_{PFN}+DIV_{SUP}\right)\times HOP_{tosink}\right)}+\beta .$$

Let Figure 7 nodes A (transmission range specified by red circle), B and C have the same number of suppressed nodes.

For Node A: H = 8, $PFN_{num}$ = 8, $HOP_{tosink}$ = 4 so $DIV_{PFN}$ = 1.

For Node B: H = 16, $PFN_{num}$ = 90, $HOP_{tosink}$ = 4 so $DIV_{PFN}$ = 15.

For Node C: H = 12, $PFN_{num}$ = 40, $HOP_{tosink}$ = 4 so $DIV_{PFN}$ = 7.

According to WDFAD-DBR, node B will be selected as a next forwarder, but it has a large number of PFNs, which will consume a lot of receiving energy and generate a large number of duplicate packets. In DOW-PR protocol, the node having highest Fitness Function (HH) value will be selected as the next forwarder. Thus, Fitness Function (HH) calculates for Nodes A, B and C as follows:

Node A:

$$HH=\frac{8}{1\times 4}=2,$$

Node B:

$$HH=\frac{16}{15\times 4}=\frac{16}{60},$$

Node C:

$$HH=\frac{12}{7\times 4}=\frac{12}{28}.$$

If source node S broadcasts a packet, then all the neighbor nodes i.e., A, B, C, M and N shown in Figure 7 acquire this packet. The suppressed nodes M and N will temporarily hold or drop the packet depending on the presence or absence of node(s) in Potential Forwarding Zone (PFZ). Nodes A, B and C are PFNs of source S and will compute the holding time and start timers. If a duplicate packet is not encountered until expiry of the timer, then this specific PFN will be selected and readily forward the packet. On the other hand, if it does not receive the duplicate, then it simply drops it. For the scenario, in which node A and B receives the packet at t1 and t2, respectively, and the duration of the packet propagated from A to B is t12. As fitness value (HH) for node A is greater than node B, then the following condition is satisfied:

(13) $$T\left[HH_{A}\right]<T\left[HH_{B}\right].$$

The holding time between two neighboring nodes should be different in such a way that the forwarder node that has a greater fitness function (HH) value transmits the packet before the transmission of the same packet from other nodes. For instance, if node A has the highest fitness function value, then it will transmit prior to node B. Upon the receiving duplicate packet from node A, it simply drops the packet. The following equation must be satisfied to avoid duplicate packets:

(14) $$t_{1}+T\left[HH_{A}\right]<t_{2}+T\left[HH_{B}\right]+t_{12}.$$

Substituting Equation (10) in Equation (13) results in:

(15) $$k\leq \frac{\left(t_{2}-t_{1}\right)-t_{12}}{HH_{A}-HH_{B}}.$$

According to the the triangle inequality theorem that the length of each side is less than sum of lengths of the other two sides, and greater than the difference between these lengths, thus $\left(t_{2}-t_{1}\right)-t_{12}$ is always less than 0, and, as $HH_{A}>HH_{B}$, thus k is always a negative number. The above two inequalities will be satisfied if:

(16) $$\left|k\right|\geq \frac{\left(t_{2}-t_{1}\right)-t_{12}}{HH_{A}-HH_{B}}.$$

For the worst case, the value of k will be:

(17) $$k=\frac{\frac{2R}{V_{0}}}{HH_{A}-HH_{B}},$$

where $V_{0}$ is the propagation speed of acoustic signal and R is the maximum transmission range. The value of *k* varies between 0 and R, as it depends on ($HH_{A}-HH_{B}$) and $HH_{A}$ ϵ [0 R]. *k* cannot always satisfy the above inequality in Equation (14) as k→−∞ when $(HH_{A}-HH_{B})\to 0$. If we replace the ($HH_{A}-HH_{B}$) by a global variable δ such that $(HH_{A}-HH_{B})\leq \delta$ , then it guarantees that node A will forward the packet before node B. Hence,

(18) $$k=\frac{\frac{-2R}{V_{0}}}{\delta }.$$

To find β, we consider that the node having the minimum Fitness Function (HH) value will have the holding time approximately zero; therefore, from Equation (10),

(19) $$\frac{\frac{-2R}{V_{0}}}{\delta }+\beta =0.$$

By solving the equation and putting the values in (10), we have:

(20) $$T\left(HH\right)=\frac{\frac{2R}{V_{0}}}{\delta }\left(R-HH\right)=0.$$

The node having the highest fitness function (HH) value will be selected as a next forwarder. For instance, node A will calculate the holding time and start timer. When the timer is expired, then node A will forward the packet. If the other nodes in the range of A, i.e., B receives the duplicate packet during the holding time, it will drop both the packets because it means the original packet is already transmitted. The holding time is inversely proportional to δ, if we select larger δ, then the holding time will decreases and therefore end-to-end delay will also reduce. Along with this improvement, there is also reduction in energy consumption that has been noticed and this is due to optimal forwarder selection at each.

### 4.9. Whale Pods Routing Protocol

Our proposed DOW-PR scheme divides the whole transmission region into two levels of nodes distribution. One level in which the nodes are in closest proximity to the surface sinks and the other is the nodes that are in closest proximity to the sink deployed underwater. There are nine sinks that are placed at the sea surface, while one is placed inside the water. The anchored nodes are fixed at the bottom that can generate and transmit a packet towards PFZ. The relay nodes are transportable with the water current in horizontal direction. These nodes are capable of generating, forwarding and receiving a packet from other nodes. Whenever the node transmits or receives a packet, the first and foremost step followed by the forwarder is to calculate its distance with the sink set D. The node compares the distance between itself and the rest of the sinks from the set D sequentially and finds PFNs lying in the hemisphere in the direction of the minimal distinct sink. The direction of data packet flow will be towards the sink lying at its closest proximity. If the separation between forwarding node and sink deployed on the sea surface is less than the sink deployed underwater, then the holding time computation will be carried out for the nodes present in the hemisphere in the direction of the surface sink D. On the other hand, if the source node is in closest proximity to the embedded sink $D_{EM}$, then the holding time computation will be carried out for the nodes present in the hemisphere in the direction of the embedded sink $D_{EM}$ .

**Figure 8:**
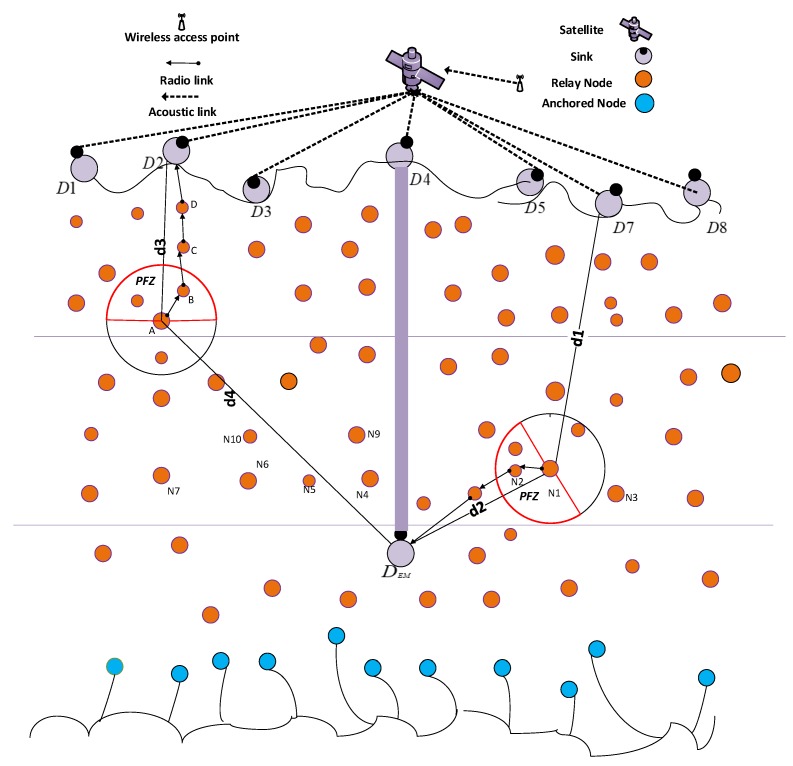
Network architecture for whale pods routing.

The above-mentioned phenomena can be further elaborated by a scenario shown in Figure 8. For example, in the network initialization phase, node N1 will first lookup for a sink in its closest proximity. For instance, after necessary computation in the initialization phase, it finds embedded sink $D_{EM}$ is the nearest sink i.e., d2<d1. Node N1 will identify the PFNs in the hemisphere centered on the virtual vector connecting it with sink $D_{EM}$. The best forwarder node will be selected using the same holding time computation described earlier in the dolphin pod technique. Likewise, if node A in Figure 8 has d4<d3, then it will find PFNs in the direction of the surface sink D. Algorithm 1 described the best forwarder selection technique i.e., valid for both dolphin pods routing and whale pods routing protocol.

**Algorithm 1:** Algorithm for selecting the forwarder among potential forwarding nodes.
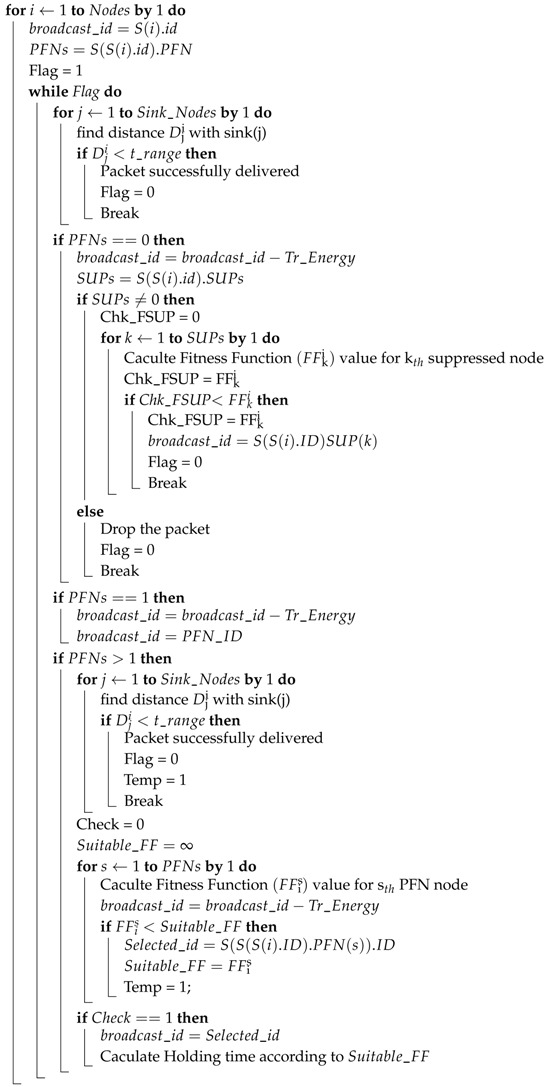


## 5. Simulation Analysis

In this section, the detailed simulation analysis of the proposed dolphin pod scheme in contrast to WDFAD-DBR is presented in addition to the simulation results in the enhanced version (whale pods routing scheme) compared with our own dolphin pods routing protocol. DOW-PR has been developed in MATLAB (Version: R2015a, Product of MathWorks, Inc.) and simulations are carried out to test the performance compared to WDFAD-DBR. We have used all the underwater environment settings as in WDFAD-DBR.

### 5.1. Simulation Setup

**Figure 9:**
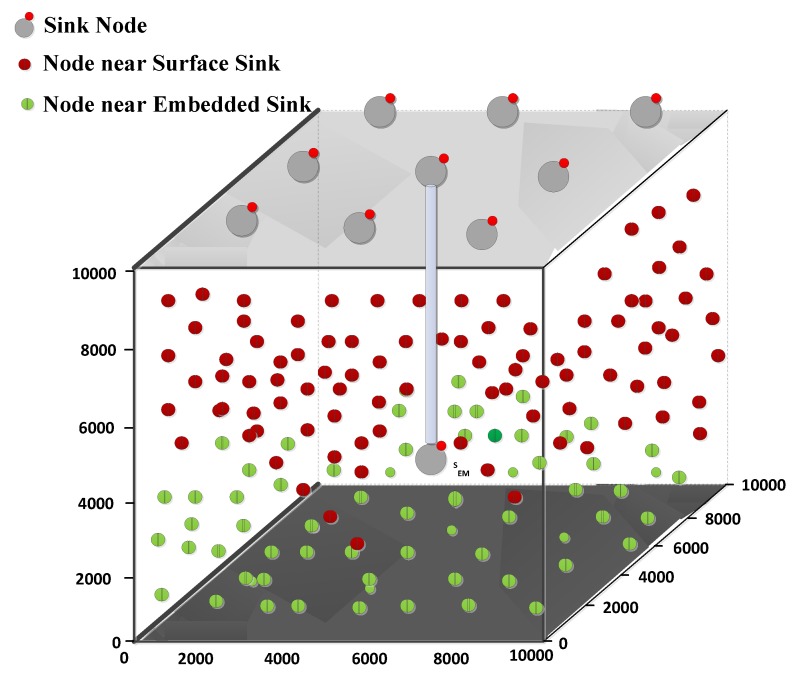
Network deployment with embedded sink $D_{EM}$.

We deploy 100–500 network nodes in the three-dimensional volumetric space of 10 Km × 10 Km × 10 Km (length, width, height), as shown in Figure 9. The average volumetric region covered by the individual node varies from 2 Km${}^{3}$ to 10 Km${}^{3}$ for dense to sparse networks respectively and if uniform network distribution is considered too. We deploy a total of nine sinks and these are fixed on the water surface. The data packet consists of header of 11 bytes while the payload size is 72 bytes with a data rate of 16 kbps. The neighbor request and acknowledgment packets length is 50 bits each. We compute the expression in Equation (9) to find the optimal forwarder among the PFNs. The parameters used in fitness function are potential forwarding nodes number $PFN_{num}$ and the suppressed nodes number $SUP_{num}$. $PFN_{num}$ and $SUP_{num}$ may have larger values in the denominator of the fitness function HH and subsequently reduces holding time difference between the PFNs and therefore may cause duplicate copies of data packet generated. To cope with the larger values of $PFN_{num}$ and $SUP_{num}$, we mapped $PFN_{num}$ and $SUP_{num}$ into arbitrary values called division factors i.e., $DIV_{PFN}$ and $DIV_{SUP}$, respectively. The main concept of these prediction arbitrary values based on the rule of thumb.These mapped values (SET1, SET2 and SET3) have been shown in Table 2 and Table 3. For instance, if we consider the 1st entry in SET3 i.e., for $1<PFN_{num}<10$ and $1<SUP_{num}<10$, the mapped values are $DIV_{PFN}$ = 1 and $DIV_{SUP}$ = 2, respectively. The simulations have been carried out for all the three sets individually and investigated the appropriate set that achieves best performance of the network. After extensive simulations, it is found that SET3 gives best results. All the simulation parameters are listed in Table 4.

### 5.2. Hop Count Mechanism

**Figure 10:**
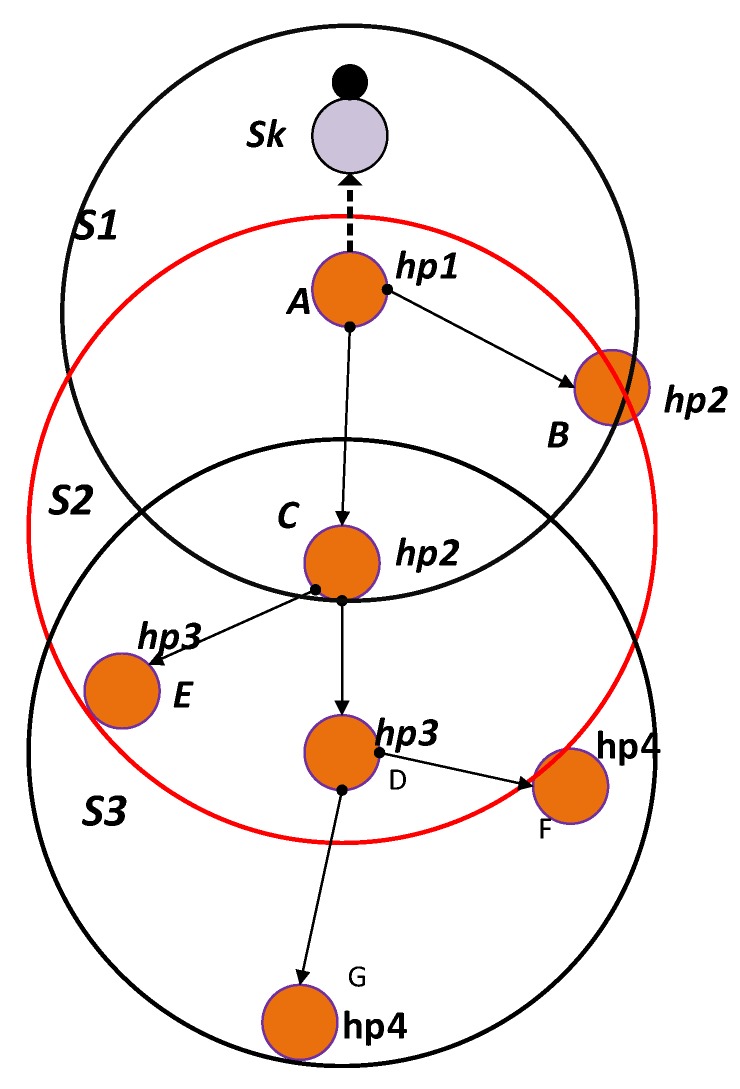
Hop count mechanism.

Hop count mechanism gives a hops number to each node according to the sequence/serial numbers of depth levels between the node and the sink. The PFNs of the source node are those nodes that have a smaller depth than the source node and lies in the range of the source node. Similarly, the nodes that are in the range of the source node but have a larger depth than the source node are called suppressed nodes. All those nodes having a sink in its range have a hop number equal to (hp1) and the nodes having depth greater than maximum transmission range PN and lesser than twice of the maximum transmission range will lie at hop 2 (hp2). Similarly, the mechanism continued until the sea bed reached. For example, from Figure 10, node A has a sink node in its range S1, so the hop number of node A is set to (hp1) . The node B and C will lie at hop 2 (hp2) if and only if these are suppressed nodes of node A and sink S1 is not in range. The node D and E will lie at hop 3 if and only if these are suppressed nodes of C and having a depth greater than twice the maximum transmission range and lesser than trice of the transmission range.

### 5.3. Data Delivery Mechanism

Data delivery mechanism is used to forward the generated or received packets to its destination. Every node can generate a packet. If the closest proximity sink is present in predefined maximum transmission power range of the source node, then it relays the packet to the sink and it is therefore successfully delivered to the destination. All the sinks are communicated/connected via high speed radio links. If the sink is not in the range, then the Algorithm 1 selects the best node for forwarding according to the fitness function (HH). The fitness function is set in such a way that the forwarding node has a higher weighting sum of depth difference of the two hops, average PFNs number, least suppressed node numbers and having a lower hops count value.

Each node is assigned a unique HH value calculated through a fitness function in Equation (9). When the HH value is set differently for each node, then, according to that, value holding time from Equation (19) for each node is calculated. The holding time is set to a very low value from Equation (12) pertaining to the node having the highest (HH) value among all the nodes. Thus, the node having the lowest holding time will bear highest priority and be selected for retransmitting the packet. All other nodes in its vicinity (range) will simply drop the packet by over hearing and therefore considerably reduce the energy consumption and the collision probability. Data delivery mechanism for Whale Pods is almost the same as Dolphin Pods, but only direction of data flow is different and it is based upon the minimum distance either from the surface sinks or embedded sink $D_{EM}$ . For instance, any node live in the region closer to the embedded sink (Figure 8) as compared to the surface sink will find next forwarder in the direction of the embedded sink. Consequently, Potential Forwarding Nodes (PFNs) will also be selected that lie in the hemisphere in the direction of the embedded sink $D_{EM}$.

## 6. Performance Comparison and Analysis

**Figure 11:**
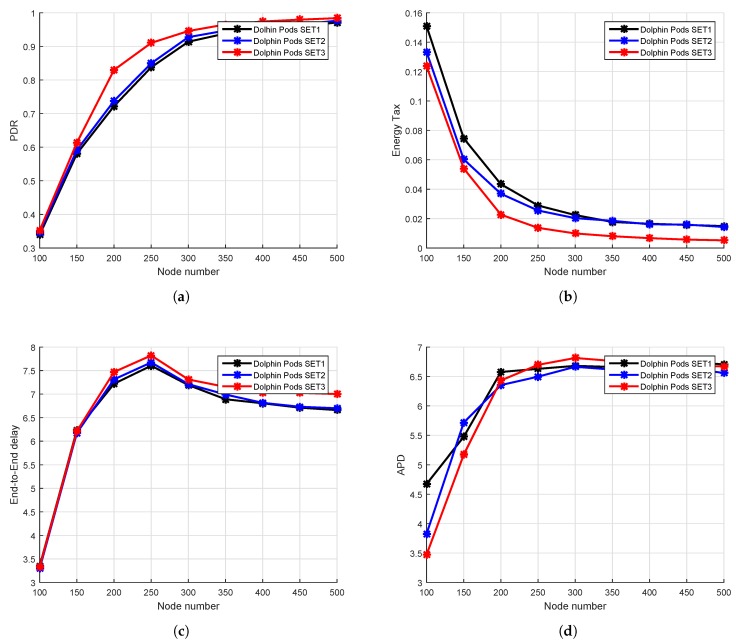
(**a**) PDR vs. number of nodes; (**b**) energy tax vs. number of nodes; (**c**) end-to-end delay vs. number of nodes; (**d**) APD vs. number of nodes. Comparison in Dolphin Pods using SET1, SET2, SET3.

We evaluate DOW-PR against WDFAD-DBR and DBR in term of APD, energy tax, end-to-end delay, packets dropped number and alive nodes number for different ranges of $PFN_{num}$ and $SUP_{num}$. We defined the measurement sets (SET1, SET2, SET3) of three different ranges for $PFN_{num}$ and $SUP_{num}$ as shown in Table 2 and Table 3, respectively. The simulation results for SET3 gives optimal results in comparison to the other two in terms of all metrics. Therefore, SET3 is found to be the best selection for simulation (refer to Figure 11).

*Performance Metrics*: The proposed system will define a new metric called Alive Nodes Number (ANN) besides the common performance metrics, i.e., APD, End-to-End Delay (E2ED), Energy Tax (ET) and PDR.

- *Alive Nodes Number (ANN)*: A node having enough energy that it can receive, process and forward the packet is called alive node. To categorize the alive nodes, the threshold $E_{TH}$ is defined i.e., the minimum energy required for the node to receive $E_{RCV}$, process $E_{PROC}$ and forward $E_{FOR}$. Threshold energy may be defined mathematically as: $E_{TH}>E_{RCV}+E_{PROC}+E_{FOR}$
- *Packet Delivery Ratio (PDR)*: PDR is the ratio of the packets received by the sink to the total packets generated by the network. The packets may be received multiple times, so this redundant packet is considered to be a one distinct packet:
  (21) $$PDR=\frac{Packets\;received}{Packet\;Sent}.$$
- *End-To-End Delay (E2ED)*: The E2ED is defined as the average time taken for a packets transmission from the instant the source node started transmission to the instant it is delivered to the destination. E2ED consists of transmission delay, propagation delay, processing time and holding time. Due to the multiple-sink nature of the network, a packet may be received by more than one sink, so the shortest time will be considered as end-to-end delay.
- *Energy Tax (ET)*: The energy tax is defined as the average energy expenditure per node when a packet is successfully delivered to its destination. It includes the energy for sending packet, receiving packet, computational energy, and the idle state energy shown in the equation below:
  (22) $$Energy\;Tax=\frac{Etotal}{nodes\times packets},$$
  where Etotal defined the total energy consumption, nodes define the total number of nodes in the network and packets define the total packets successfully received by the sink. The duplicate packet received by the sink is removed from the total number of packets because energy tax is the amount of energy per packet per node in the network.
- *Average Accumulated Propagation Distance (APD)*: APD is defined as the average accumulated distance of each hop of all the packets that are successfully delivered to the sinks. There is a multi-sink network environment in which more than one sink can receive a packet, so the shortest accumulated propagation distance is considered as a final accumulated propagation distance.
  The APD can be found by the following mathematical equation:
  (23) $$APD=\frac{1}{n\_p}\sum_{j=1}^{n_{p}}\sum_{i=1}^{h}dist_{j}^{i},$$
  where $n_{p}$ is the number of packets, *h* is the hop number of packet from source node to sink and $dist_{j}^{i}$ is the distance of the ith hop of the jth packet.

### 6.1. Simulation Results in the Dolphin Pods Routing Scenario

**Figure 12:**
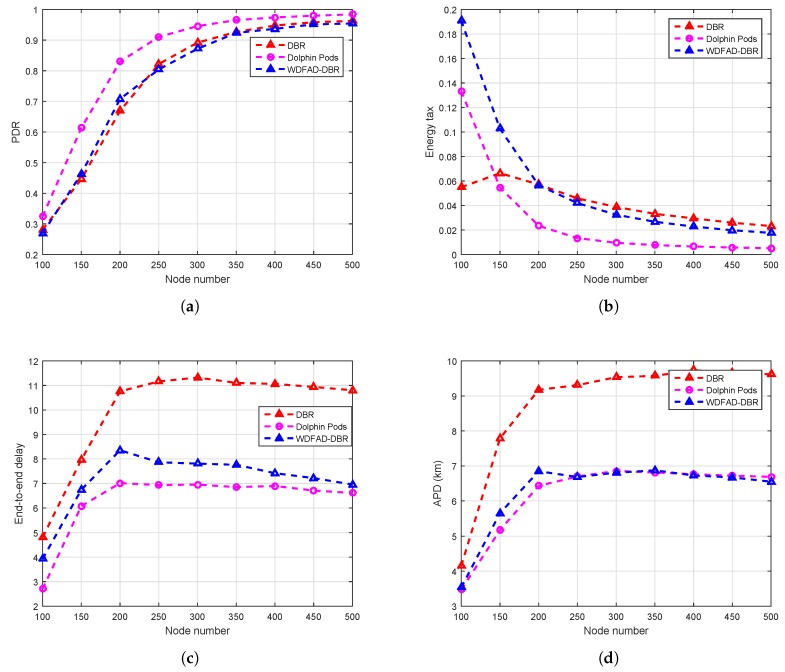
(**a**) PDR vs. number of nodes; (**b**) energy tax vs. number of nodes; (**c**) end-to-end delay vs. number of nodes; (**d**) APD vs. number of nodes. Simulation results using arbitrary values in SET3.

In this section, the simulations are performed upon the dolphin pod routing in which all the sinks are placed on the water surface without any sink embedded $D_{SM}$ underwater. It can be seen from Figure 12 that the similar trend of increasing PDR for DBR, WDFAD-DBR and DOW-PR protocol when network density increases. The reason behind the common trend is the fact that, if density of the network increases, there will be more probability of occurrence of active node(s) at the next hop and therefore a reduction of void holes. DOW-PR outperformed in terms of PDR in comparison to WDFAD-DBR and DBR. In DBR, the node having the lowest depth among the potential forwarding nodes will be selected as a next forwarder and will not consider the depth of the expecting hop, which results in increasing the chance of a void hole. However, WDFAD-DBR selects the next expecting hop on the basis of weighting sum of depth difference of the two hops, which reduces the chance of a void hole happening. The PDR of DBR and WDFAD is almost the same from node numbers 270–500, i.e., in a dense network. The reason is the presence of enough PFNs in the range of a source node, which reduces the probability of void hole. PDR of a dolphin pod is higher than WDFAD-DBR, primarily because WDFAD-DBR selects the next expecting hop on the basis of weighting sum of depth difference between the two hops. Nonetheless, a dolphin pod considers all the factors including weighting sum of depth difference of the two hops, the number of PFNs, suppressed nodes number, and the hop count value to sink. The difference between the PDR of dolphin pod and WDFAD is higher for a sparse network and reduces due to the dense network. Secondly, WDFAD-DBR drops the packet in the absence of PFN, but dolphin pods select a node for forwarding from the suppressed nodes and therefore prevent the loss of the data. The PDR of dolphin pods is higher than WDFAD-DBR in both sparse and dense networks. However, in a sparse network, WDFAD-DBR more ofen drops the packet due to high probability of void holes and therefore a more proportional gain of PDR in dolphin pods resulted. On the other hand, as node number increases and void hole probability decreases, the fraction gap in PDR results. Moreover, there are two types of void holes occurring in routing protocols. One is due to lack of a potential forwarder in the range of a source node and the other is due to the lack of energy in a potential forwarding node [34]. This means that there is a forwarding node of the source node but it doesn’t have a sufficient threshold energy. The dolphin pod is trying to avoid both the void and energy holes. When the void holes occurred due to no PFN in the range of the source node, it selects a forwarding node from the suppressed nodes as shown in Figure 6. Subsequently, it reduces the re-transmissions and reduces the energy consumption due to redundant packets’ avoidance. When the energy consumption is reduced, then the energy tax or an average energy expenditure per packet of each node is decreased as it is clear from Equation (22). Consequently, energy is conserved and therefore the occurrence of energy holes is also reduced. As a result, by overcoming both the void and energy hole, the PDR of the dolphin pods’ routing scheme increases. The PDR statistics are shown in Table 5, in which we can easily notice the PDR improvement by 11.89%, 6.085% and 3.365% in the scenario where node densities are 200, 300 and 400, respectively.

Moreover, dolphin pod routing assigns weight both to the packet advancement as well as to the network traffic density in such a way that priority is given to the less dense traffic path at the cost of packet advancement. The fitness function (HH) value will be less for the more dense path in which the probability of traffic density is high. Therefore, the dolphin pods scheme selects the path where the forwarder of the source node has a higher value of weighting sum of depth difference of the hops (H) [3], average number of forwarding nodes, very small number of suppressed nodes and is close to the sink, which means that the fitness function (HH) for that path is greater, which reduces the collision probability at the receiver. As a result, the PDR value is increased.

Next, we investigate the energy tax comparison of a proposed dolphin pod with WDFAD-DBR protocol. When compared to idle listening, packet reception, sensing and processing of operations, in underwater acoustic networks, transmission of a packets is the most energy consuming operation. Transmission of data packets accounts for most of the energy consumed due to their large size when compared to control packets. The above argument is already validated through experimental measurement by the authors in [39]. Energy cost for transmitting a single data bit is roughly equal to the energy consumption for processing thousands of operations [40]; however, complexity of the algorithm may increase energy cost. The algorithm we proposed considers all of the above-mentioned energy usage parameters. However, only considering the receiving and transmitting energies will also not affect the general trend. DBR and WDFAD-DBR do not exploit certain energy efficient parameters, due to which our proposed scheme (DOW-PR) outperformed the two in terms of energy conservation. The simulation results are drawn for energy tax against the nodes number in the network (refer to Figure 11 for mapping into arbitrary values in SET3). The similar trend found for energy tax in all protocols DBR, WDFAD-DBR, and dolphin pod i.e., energy consumption reduces when the nodes number increases. This is due to the fact that increasing nodes in the network causes the increase of energy resource and also the probability of successful packet delivery being improved. Therefore, it reduces the retransmissions of the packets as nodes number increases and will significantly reduce the energy wastage.

Secondly, our scheme exploited the adaptive nature of data transmission power, which depends upon the maximum displaced node within the transmission range (refer to Figure 5). Consequently, a significant amount of energy saving resulted, in contrast to WDFAD-DBR in which a fixed amount of energy was utilized at each hop regardless of the node displacement. In sparse networks, the transmission power adjustment is not very effective due to the nodes being widely dispersed, and there will be low probability of a nearby potential forwarder for lesser transmission energy usage. On the other hand, in dense networks, there will more likely be the presence of nodes in the maximum transmission range and therefore more options of ranges can be investigated. Generally, source nodes need to transmit with maximum power in case there is a forwarding node in its farthest transmission zone. Consequently, a greater number of nodes will receive the packet due to maximum transmission, but this is a rare occurrence. However, in WDFAD-DBR, the source node will transmit with a fixed maximum power level, no matter if the farthest forwarding node is even lying in a close proximity transmission zone. The above procedure adopted in our proposed scheme significantly reduces the energy consumption without compromising other performance parameters. Therefore, either being sparse or dense, the proposed protocol (DOW-PR) convincingly beat both DBR and WDFAD-DBR in terms of efficient energy utilization.

Moreover, the proposed scheme in this paper selects the next expecting hop by considering the weighting sum of the depth difference of the two hops, the PFN number, suppressed nodes number and the hop number of the expected next forwarding node, which reduces the total energy to a very low level. Energy tax is directly proportional to the total energy consumption, and inversely related to nodes number and number of packets generated (refer to Equation (21). WDFAD-DBR selects the next forwarding node by taking the accumulative advancement between the two hops, but it does not consider the receiving energy consumption due to available PFNs number and suppressed nodes number. Dolphin pod considers the energy efficient forwarder/path based upon the receiving energy consumption in potential forwarding nodes number and suppressed nodes number associated with the source node. Dolphin pod gives weight to both parameters by setting the division factors $DIV_{PFN}$ and $DIV_{SUP}$. If the number of PFNs and SUP nodes number are less, then the division factors ($DIV_{PFN}$ and $DIV_{SUP}$) are set to very low values and, therefore, the receiving energy consumption in this case is negligible. Consequently, the forwarder selection criteria will then only be based on the weighting sum of depth difference of the hops (H) value as from Equation (10). For the case, if number of PFNs and number of suppressed nodes of a PFN are greater, dolphin pods set the division factors to a high value and will then be based on number of PFNs and suppressed nodes number means the receiving energy is consumed in larger amounts, so the forwarder/path, which has a low value of fitness function, will be selected.

It can also be easily judged through Figure 12b in which there is huge reduction in energy tax. However, the percentage improvement in energy tax reduces as nodes number increases. This is due to the fact that the collision probability increases at the receiver, and the number of retransmissions will consume quite a lot more energy. The analysis shows that there are 37.07%, 30.81%, 29.11% and 25% more energy conserved for 200, 300, 400 and 500 nodes, respectively (refer to Table 6).

Primarily, end-to-end delay increases for both dolphin pod routing and WDFAD-DBR protocol by increasing the nodes density i.e., from 100 to 250 nodes. For any further increase in node density, the end-to-end delay appears to be reduced, and this is due to number of reasons that included reducing hops count, increasing collision probability and better successful packets delivery at the destination. In a dense network scenario, there are enough nodes readily available at the edge of the transmission range for selecting the next hop forwarding node. Dolphin pod considers the hop number of each node in fitness function, which reduces the APD as well as end-to-end delay. The analysis shows that there are 37.07%, 30.81%, 29.11% and 29.00% average improvement in end-to-end delay for 200, 300, 400 and 500 nodes, respectively (refer to Table 7).

**Figure 13:**
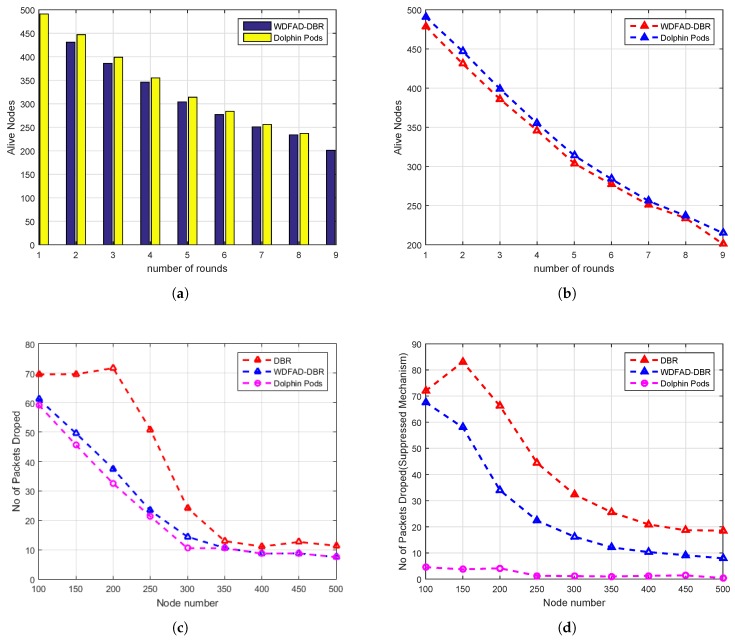
(**a**) number of alive nodes vs. rounds; (**b**) number of alive nodes vs. rounds; (**c**) number of packets dropped with suppressed vs. number of nodes; (**d**) number of packets dropped without suppressed vs. number of nodes. Simulation results using arbitrary values in SET3.

Along with the above-mentioned improvements in the performance metrics, we also investigate the other important metric, the average number of packets dropped in the network. Referring to Figure 13, it can be easily noticed that there is a significant reduction of packet drop in the proposed DOW-PR scheme as compared to WDFAD-DBR.

The reasoning behind this improvement is that our mechanism ensures better life span of the individual local nodes and the network as well. The logical arguments are somehow similar to energy tax improvement described earlier.

WDFAD-DBR does not take into account the void hole occurrence probability; instead, it only considered the packet upward distance advancement at the two hops. On the other hand, the proposed DOW-PR scheme considered the potential forwarder nodes number at both one and two hops. Moreover, if the source node does not find the forwarder in the forwarding direction, then it could select a node from the suppressed region and therefore the scheme came up with utmost reduction of average packets dropped by a nodes in the network (refer to Figure 13c). The logic not only causes the reduction of the packets dropped number, but it also significantly improved the energy consumption. The result shown in the Figure 13a,b for alive nodes number against the number of simulation rounds. It has been noticed that, as number of rounds increases, the number of alive nodes reduces.

We further elaborate the above-mentioned trend in Figure 13c that the number of packets drop reduces with the increase in network density. This is due to the fact that there are more numbers of alternative forwarders readily available in the dense networks compared to sparse networks. Consequently, the number of packets dropped reduces. The other strong reason is that, in a sparse network, the packets may not reach the distinct neighbors due to high bit error rate or degraded link quality, and hence the packets dropped. Conversely, in dense networks, enough nodes are placed in close vicinity of the source node, which causes reduction of packets being dropped. However, the hops count value to reach the destination increased and may degrade end-to-end delay.

### 6.2. Simulation Results in the Whale Pods Routing Scenario

**Figure 14:**
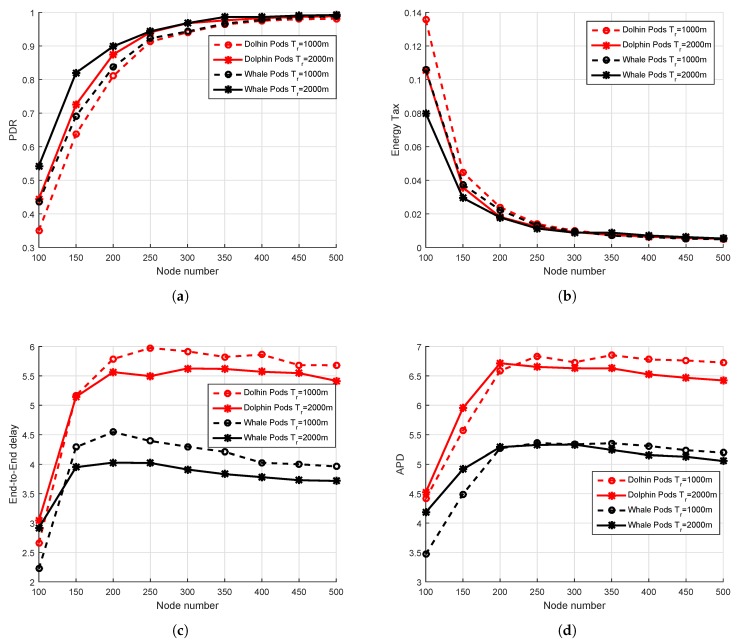
(**a**) PDR vs. number of nodes; (**b**) energy tax vs. number of nodes; (**c**) end-to-end delay vs. number of nodes; (**d**) APD vs. number of nodes. Comparison of dolphin pods with whale pods/routing.

The simulations have been repeated for the whale pod routing version of the proposed DOW-PR protocol. It has been shown in the results that there is a great deal of improvement compared to its predecessor dolphin pod routing in all the prescribed performance metrics. Referring to Figure 14b, it can be observed that the energy tax of the whale pod DOW-PR protocol is reduced throughout the density of the network (i.e., from 200 to 500 nodes). The reasons behind this is that the packet(s) generated/forwarded from the nodes in the vicinity of the embedded sink does not have to travel a long distance. Instead, it is collected locally. This means that the few forwarders involved have a lesser amount of energy consumed.

It can also be easily judged through Figure 14d in which there is huge reduction in average APD and therefore reduces the number of hops propagated. However, the percentage improvement in energy tax reduces as nodes number increases. This is due to the fact that the collision probability increases at the receiver and number of retransmissions will consume quite a lot more energy.

Moreover, it is further analyzed that there are more number of copies generated or forwarded as the messages pass through more hops. Next, the deployment of an additional sink in the whale pod scenario causes a significant improvement in the PDR (refer to Figure 14a). It can be seen that our updated scheme i.e., whale pod routing protocol beats the dolphin pod routing regardless of any number of nodes in the network. Behind this improvement, there are multiple reasons that include the high probability of finding the sink in the range of nodes, less number of hops count, less propagation distance and so on. The closest proximity nodes to the embedded sink either directly communicate with the sink or through a fewer number of hops and therefore the probability of packets drop reduces.

Last but not least is the important metric end-to-end delay that needs to be optimized especially when there is run-time data needed in the network. The end-to-end delay has been investigated for both DOW-PR scenarios. The simulation results show the obvious improvement in end-to-end delay and this is because of the same argument described repeatedly in the above text i.e., the locally available embedded sink reduces the propagation distance. For instance, if the node at the seabed generated a packet, then it has to propagate at least the full *z*-coordinate (i.e., 10 km) to be collected by the sink at the sea surface. On the other hand, in the whale pod scenario, the same packet will be received at the embedded sink and propagated through the distance of 2.5 km at the most.

Although our proposed protocol outperforms the aforementioned performance metrics, we observe some limitations and constraints in certain specific scenarios as discussed below:

In sparse networks, there are probably fewer PFNs present at each node in the network due to which overall improvement seems to be negligible; however, the computational cost still becomes greater. The abovementioned constraint in DOW-PR will become even more pressing when transmission range is also low. WDFAD-DBR focuses on the packet advancement at the first and second hop. It works well compared to DOW-PR in delay intolerant ad hoc underwater networks, where prolonged network lifetime is not required. DOW-PR has other limitations in the scenario where the source node is in a void and its predecessors also don’t have PFNs other than the source node. In this case, the node will drop the packet or have progressive backward transmissions. In sparse networks with low average data packet generation, a low number of duplicate packets occurs due to which energy losses reduce in both DOW-PR and WDFAD-DBR, but DOW-PR will carry out more complex computational algorithms. In sparse networks, the probability of the void hole occurrence is high due to which DOW-PR will produce a huge number of void announcement messages, which will be disseminated in the entire network. It has been observed that, contrary to WDFAD-DBR, DOW-PR undergoes additional energy loss in transmitting void announcement control packets and thereby collision probability is also increased. In the future, the authors plan to undertake research that will overcome the above-mentioned constraints.

## 7. Conclusions

A dolphin and whale pods routing protocol (DOW-PR) has been proposed in this paper. There are two versions of the proposed protocols i.e., dolphin pods and the whale pods. Whale pods routing protocol is the further enhancement of the dolphin pods routing. Basically, the proposed scheme enhanced the performance of the metrics simulated in its state-of-the-art counterpart routing protocol i.e., Weighting Depth Forwarding Area Division Depth Based Routing (WDFAD-DBR) protocol. DOW-PR provides a reduction in energy consumption, increasing PDR, and minimizing end-to-end delay. In the proposed scheme, it explores the role of the parameters like potential forwarding nodes number, suppressed nodes numbers and hops count value in designing the holding time equation. Moreover, the values of the above-mentioned parameters have been mapped (SET1, SET2 and SET3) into arbitrary values and that are also tested for simulations. The investigations through simulations showed that dolphin pods routing beats the WDFAD-DBR, on average improving by 31.5% of energy tax, 6% of PDR, and 24.6% of E2ED. However, certain performance trade-offs have been observed and listed in Table 8. Furthermore, the whale pods routing protocol outperformed the dolphin pod version and therefore improvement is seen in energy tax, PDR and E2ED.

In the future, we will investigate the other sets of arbitrary values for mapping the actual values of potential forwarding nodes number, suppressed nodes number and hops count. Other than this, we will deploy a greater number of movable underwater sink nodes.

## Figures and Tables

**Table 1:** Qualitative Comparison of UWSN protocols.

Protocol	Delivery Ratio	Delay Efficiency	Energy Efficiency	Bandwidth Efficiency	Reliability	Cost Efficiency	Performance
VBF [9]	Low	Low	Fair	Fair	Low	n/a	Low
HH-VBF [11]	Fair	Fair	Low	Fair	High	n/a	Fair
ESEVBF [20]	High	Low	High	Fair	High	n/a	High
FBR [10]	Fair	High	High	Fair	Fair	n/a	High
DFR [13]	Fair	Fair	Low	Fair	High	n/a	Fair
Mutipath [15]	Fair	Fair	Low	Fair	High	Low	Fair
DBR [2]	High	High	Low	Fair	High	Fair	High
WDFAD [3]	High	Low	Fair	Fair	High	Fair	High
DOW-PR	High	Fair	High	Fair	High	Fair	High
Adaptive [7]	High	Fair	Flexible	Flexible	Flexible	n/a	Fair
ICRP [19]	Fair	Low	Fair	Fair	Low	High	Low
DUCS [17]	Fair	Low	Fair	Fair	Low	High	Low
HydroCast [18]	High	High	Fair	Fair	Fair	Fair	High
MCCP [10]	Low	Low	High	Fair	Fair	High	Fair
H2-DAB [31]	High	Fair	Fair	Fair	Fair	High	Fair

n/a: not applicable

**Table 2:** Actual number of PFNs mapped into arbitrary values.

SET1		SET2		SET3	
**Actual Range**	**Arbitrary Value**	**Actual Range**	**Arbitrary Value**	**Actual Range**	**Arbitrary Value**
1 $\leq PFN_{num}\leq$ 100	$DIV_{PFN}$ = 1	1 $\leq PFN_{num}\leq$ 50	$DIV_{PFN}$ = 1	1 $\leq PFN_{num}\leq$ 10	$DIV_{PFN}$ = 1
100 $\leq PFN_{num}\leq$ 200	$DIV_{PFN}$ = 2	50 $\leq PFN_{num}\leq$ 100	$DIV_{PFN}$ = 2	10 $\leq PFN_{num}\leq$ 25	$DIV_{PFN}$ = 3
200 $\leq PFN_{num}\leq$ 300	$DIV_{PFN}$ = 3	100 $\leq PFN_{num}\leq$ 200	$DIV_{PFN}$ = 4	25 $\leq PFN_{num}\leq$ 50	$DIV_{PFN}$ = 7
300 $\leq PFN_{num}\leq$ 400	$DIV_{PFN}$ = 4	200$\leq PFN_{num}\leq$ 450	$DIV_{PFN}$ = 7	50 $\leq PFN_{num}\leq$ 100	$DIV_{PFN}$ = 15
400 $\leq PFN_{num}\leq$ 500	$DIV_{PFN}$ = 14	450 ≤ $\leq PFN_{num}\leq$ 500	$DIV_{PFN}$ = 11	100 $\leq PFN_{num}\leq$ 200	$DIV_{PFN}$ = 25
-	-	-	-	200 $\leq PFN_{num}\leq$ 350	$DIV_{PFN}$ = 39
-	-	-	-	350 $\leq PFN_{num}\leq$ 500	$DIV_{PFN}$ = 75

**Table 3:** Actual number of SUPs mapped into Arbitrary Values.

SET1		SET2		SET3	
**Actual Range**	**Arbitrary Value**	**Actual Range**	**Arbitrary Value**	**Actual Range**	**Arbitrary Value**
1 $\leq SUP_{num}\leq$ 100	$DIV_{SUP}$ = 5	1 $\leq SUP_{num}\leq$ 50	$DIV_{SUP}$ = 2	1 $\leq SUP_{num}\leq$ 10	$DIV_{SUP}$ = 2
100 $\leq SUP_{num}\leq$ 200	$DIV_{SUP}$ = 8	50 $\leq SUP_{num}\leq$ 100	$DIV_{SUP}$ = 5	10 $\leq SUP_{num}\leq$ 25	$DIV_{SUP}$ = 5
200 $\leq SUP_{num}\leq$ 300	$DIV_{SUP}$ = 9	100 $\leq SUP_{num}\leq$ 200	$DIV_{SUP}$ = 11	25 $\leq SUP_{num}\leq$ 50	$DIV_{SUP}$ = 11
300 $\leq SUP_{num}\leq$ 400	$DIV_{SUP}$ = 11	200 $\leq SUP_{num}\leq$ 350	$DIV_{SUP}$ = 15	50 $\leq SUP_{num}\leq$ 100	$DIV_{SUP}$ = 22
400 $\leq SUP_{num}\leq$ 500	$DIV_{SUP}$ = 14	450 $\leq SUP_{num}\leq$ 500	$DIV_{SUP}$ = 21	100 $\leq SUP_{num}\leq$ 200	$DIV_{SUP}$ = 41
-	-	-	-	200 $\leq SUP_{num}\leq$ 300	$DIV_{SUP}$ = 65
-	-	-	-	300 $\leq SUP_{num}\leq$ 350	$DIV_{SUP}$ = 75
-	-	-	-	350 $\leq SUP_{num}\leq$ 500	$DIV_{SUP}$ = 87

**Table 4:** Parameters’ settings.

Parameters	Values
Number of nodes	100:50:500
Number of sinks	9
Maximum transmission range of each node	2 km
Deployment region: 3D Region of 10 Km	Length: 10 km
-	Height: 10 km
-	Width: 10 km
Header size of DATA	11 Bytes
Payload size OF DATA	72 Bytes
Size of ACK Packet	50 bits
Size of Neighbor Request	50 bits
Data rate	16 Kbps
Initial Energy of each node	100 J
Maximum transmission power	90 dB re μPa
Power threshold for receiving	10 dB re μPa
Sending Energy	50 W
Receiving Energy	158 mW
Idle Energy	158 mW
Center Frequency	12 KHz
Acoustic Propagation	1500 m/s
δ	2 Km
Bandwidth	4 KHz
Random Walk	2 m/s
Probability of moving left	0.5
Probability of moving right	0.5
Alive node threshold energy	5 W

**Table 5:** Overall PDR improvement in dolphin pods routing compared to WDFAD-DBR.

Node Number	200	300	400	500
Improvement with Tr = 1000 m	14.61%	7.12%	4.23%	3.56%
Tr = 2000 m	9.17%	5.05%	2.5%	1.5%
Average Improvement	11.89%	6.085%	3.365%	2.53%

**Table 6:** Overall Energy saved in dolphin pods routing compared to WDFAD-DBR.

Node Number	200	300	400	500
Improvement with Tr = 1000 m	38.46%	30.12%	28.32%	28.49%
Tr = 2000 m	35.68%	31.50%	29.90%	29.51%
Average Improvement	37.07%	30.81%	29.11%	29.00%

**Table 7:** Overall End-to-End delay improvement of dolphin pods with WDFAD-DBR.

Node Number	200	300	400	500
Improvement with Tr = 1000 m	24.76%	24.11%	22.50%	20.12%
Tr = 2000 m	31.17%	26.89%	24.62%	23.17%
Average Improvement	27.96%	25.5%	23.56%	21.64%

**Table 8:** Performance trade-offs.

Features	Achievements	Price to pay
Forwarder selection from SUPs	PDR improvement Figure 12a	End-to-end delay Figure 12c
Prioritizing forwarder through Holding time	Energy consumption reduction Figure 12b	Upward packet advancement
	Duplicate packets reduction		
	Collision avoidance		
Embedded Sink	Prolong network lifetime Figure 13c,	Deployment and physical
	end-to-end delay reduction Figure 14c,	connection maintenance cost
	Reliability Figure 14a	
Transmission zones	Energy tax reduction Figure 12b	Computationally complex
Hop Count Mechanism	APD Improvement Figure 12d	Computationally complex
	end-to-end delay Figure 12c		
